# Efficacy of Acupuncture versus Combined Oral Contraceptive Pill in Treatment of Moderate-to-Severe Dysmenorrhea: A Randomized Controlled Trial

**DOI:** 10.1155/2015/735690

**Published:** 2015-08-04

**Authors:** Intira Sriprasert, Suparerk Suerungruang, Porntip Athilarp, Anuchart Matanasarawoot, Supanimit Teekachunhatean

**Affiliations:** ^1^Department of Obstetrics and Gynecology, Faculty of Medicine, Chiang Mai University, Chiang Mai 50200, Thailand; ^2^Health Promotion Center Region 10, Department of Health, Ministry of Public Health, Chiang Mai 50100, Thailand; ^3^Center of Thai Traditional and Complementary Medicine, Faculty of Medicine, Chiang Mai University, Chiang Mai 50200, Thailand; ^4^Department of Family Medicine, Faculty of Medicine, Chiang Mai University, Chiang Mai 50200, Thailand; ^5^Department of Pharmacology, Faculty of Medicine, Chiang Mai University, Chiang Mai 50200, Thailand

## Abstract

This open-label randomized controlled trial was designed to compare the efficacy of acupuncture and combined oral contraceptive (COC) pill in treating moderate-to-severe primary dysmenorrhea. Fifty-two participants were randomly assigned to receive either acupuncture (*n* = 27) or COC (*n* = 25) for three menstrual cycles. Mefenamic acid was prescribed as a recue analgesic drug with both groups. The statistical approach used for efficacy and safety assessments was intention-to-treat analysis. By the end of the study, both treatments had resulted in significant improvement over baselines in all outcomes, that is, maximal dysmenorrhea pain scores, days suffering from dysmenorrhea, amount of rescue analgesic used, and quality of life assessed by SF-36 questionnaire. Over the three treatment cycles, COC caused greater reduction in maximal pain scores than acupuncture, while improvements in the remaining outcomes were comparable. Responders were defined as participants whose maximal dysmenorrhea pain scores decreased at least 33% below their baseline. Response rates following both interventions at the end of the study were not statistically different. Acupuncture commonly caused minimal local side effects but did not cause any hormone-related side effects as did COC. In conclusion, acupuncture is an alternative option for relieving dysmenorrhea, especially when COC is not a favorable choice.

## 1. Introduction

Dysmenorrhea is one of the most common gynecologic problems affecting women of reproductive age with a prevalence of 50–90% [1–3]. One-third of women reported moderate-to-severe pain which impacts the quality of life as it leads to absence from school or work as well as decreased social interest and participation [4]. Dysmenorrhea can be categorized as either primary dysmenorrhea which occurs without demonstrable disease or secondary dysmenorrhea which involves underlying pathology such as endometriosis, adenomyosis, or uterine myoma. Dysmenorrhea in young women is mostly primary dysmenorrhea, with the prevalence decreasing with age. The pathogenesis of primary dysmenorrhea is explained by the action of prostaglandins such as prostaglandin E2, prostaglandin F2*α*, endoperoxides, or vasopressin released from the endometrium at the onset of menstruation which stimulates pain neurons and induces excessive contraction of the uterus and adjacent organs causing ischemia which is perceived as pelvic pain [5]. Beside pelvic pain, some women experience associated symptoms such as nausea, vomiting, headache, migraine, dizziness, diarrhea, constipation, fatigue, or lightheadedness.

A nonsteroid anti-inflammatory drug (NSAID) is suggested for dysmenorrhea pain relief as it inhibits activity of cyclooxygenase and thereby the synthesis of prostaglandins which are believed to cause dysmenorrhea [6]. A combined oral contraceptive (COC) pill is also recommended for the treatment of primary dysmenorrhea due to its ability to suppress ovulation and reduce menstrual prostanoids [5], providing relief from dysmenorrhea pain in up to 70–80% of women [7–10]. A COC containing low-dose ethinyl estradiol and third generation progestin usually shows a decrease in severity of dysmenorrhea after 3 to 6 treatment cycles [10]. However, even with the use of an NSAID and COC, 25% of women with primary dysmenorrhea have reported no response [11, 12] or treatment-related side effects such as nausea, headaches, and weight gain [8, 13].

Acupuncture is a complementary therapy involving the insertion of fine needles into the body at specific points to achieve a therapeutic effect [14]. Acupuncture has been reported to be successful in treating primary dysmenorrhea [15, 16]. According to acupuncture theory, syndrome differentiation of dysmenorrhea can be divided into “Excess” type (i.e., stagnation of vital energy and blood, stagnation of cold, and accumulation of damp-heat) and “Deficiency” type (which is further subdivided into two categories: one is vital energy and blood deficiency and the other is kidney and liver deficiency). Acupuncture's primary purpose is to move vital energy and blood, scatter cold, clear damp-heat, or tonify/nourish vital energy, blood, and internal organs with respect to the syndrome differentiation. On a scientific basis, acupuncture causes the central nervous system to release endorphins, serotonin, and acetylcholine which results in pain reduction [15]. A systematic review concluded that acupuncture provides a reduction in dysmenorrhea pain when compared to a placebo control [12]. In addition to pain relief, acupuncture has been reported to improve quality of life and decrease NSAID use after regular treatment for 3 to 6 months [16, 17].

Although several studies have reported benefits from acupuncture treatment for dysmenorrhea [12, 15–17], no head-to-head comparative study of the relative efficacy of acupuncture and COC, especially in women with moderate-to-severe primary dysmenorrhea, has been conducted. This study aimed at investigating the relative efficacy of acupuncture and COC in this clinical setting.

## 2. Materials and Methods

### 2.1. Design

This prospective, open-label, randomized controlled trial was conducted during the period from January 2013 to February 2014 at the Center of Thai Traditional and Complementary Medicine (TTCM), Faculty of Medicine, Chiang Mai University, Chiang Mai, Thailand. It was approved by the Research Ethics Committee of the Faculty of Medicine, Chiang Mai University. This study was also registered with the Chinese Clinical Trial Registry (http://www.chictr.org/, registration number: ChiCTR-TRC-13003236).

### 2.2. Sample Size Calculation

Calculation of sample size was based on the assumption that a percentage of responders in each intervention group would be the main criterion for measuring efficacy and that the percentages of responders in the acupuncture group and the COC group would be 65% [17] and 85% [13], respectively. Additionally, the noninferiority margin was assumed to be 15%. To establish one-sided noninferiority of two parallel-sample proportions [19–21], the required sample size to achieve 80% power (*β* = 0.2) at *α* = 0.05 for detecting such a difference was 18 participants per group. With a projected dropout rate of 30%, at least 24 participants per treatment group or a total sample size of at least 48 participants was needed. Fifty-two individuals met the eligibility criteria and were randomized into one of the two treatment groups in this study.

### 2.3. Participants

Women aged between 18 and 35 years were recruited from Chiang Mai province, Thailand. During a run-in period of one month, participants were instructed to discontinue all treatments for dysmenorrhea with the exception of the rescue analgesic drug prescribed by the investigator (see below). After the run-in period, participants who met the eligibility criteria were randomly assigned to receive one of two interventions, either COC pill or acupuncture. Inclusion criteria were a history of dysmenorrhea within the previous three consecutive months with a numeric rating scale (NRS) of 5 or more, a verbal multidimensional scoring system (VMSS) of grade 2 or more, and the use of rescue analgesic drug for dysmenorrhea (at least 1 tablet during the run-in period). Exclusion criteria were contraindications to COC or acupuncture and underlying gynecologic conditions associated with dysmenorrhea. Transabdominal ultrasonography was performed by a gynecologist to rule out abnormal gynecologic conditions such as uterine myoma, endometrioma, and ovarian tumor. All participants were informed about the study and gave written consent prior to participation.

### 2.4. Interventions

After screening, the participants who met the eligibility criteria were randomly assigned to receive either COC pills (the COC group) or acupuncture (the ACU group) for three consecutive menstrual cycles. The randomization list was generated using a computer program. The allocation process was accomplished by placing the allocation cards in individual sealed envelopes to preserve concealment. The envelopes were then numbered and given to the participants after they had successfully completed all baseline assessments. All participants in the study received a supply of rescue NSAID, mefenamic acid (250 mg per tablet), prescribed at 1 tablet as needed for dysmenorrhea to be taken based on the perception of pain by patients themselves and to be repeated every 4 to 6 hours in case of no significant dysmenorrhea pain relief.

The COC group participants were treated with a daily monophasic COC containing 20 *µ*g ethinyl estradiol and 150 *µ*g desogestrel (Mercilon MSD, manufactured by Merck Sharp & Dohme, 21 active pills and 7 placebo pills per cycle). They were instructed to take the first pill within five days after the first day of their last menstrual period (LMP) and continue taking one pill every day for three cycles. A pill count was conducted at every follow-up visit to assess compliance.

**Figure 1:**
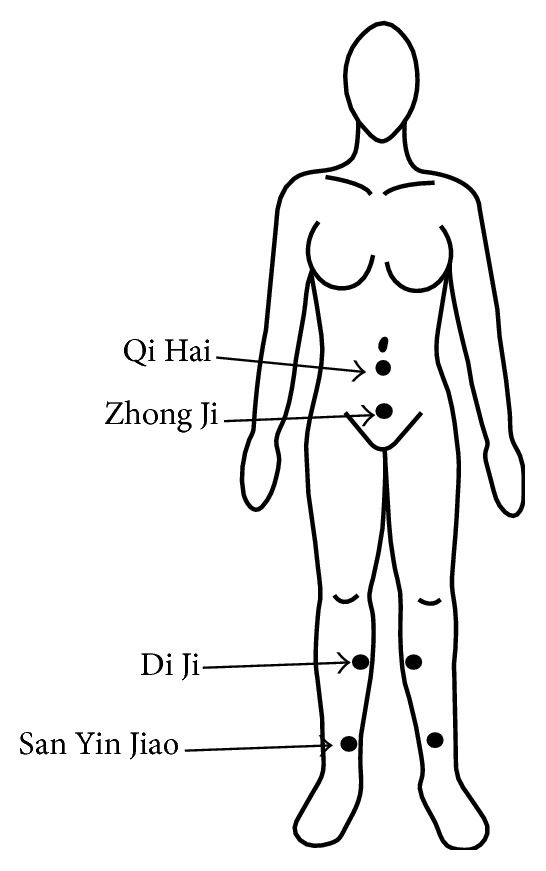
Acupuncture points used in this study.

In the ACU group, the acupuncture protocol used in this study was an acupuncture formula consisting of six acupuncture points per session (Figure 1 and Table 1). The needles were inserted in the following order: Qi Hai (Ren 6), Zhong Ji (Ren 3), and both sides of Di Ji (SP 8) and San Yin Jiao (SP 6). Six sterile acupuncture needles (size 0.25 × 40 mm) were inserted into each point to the appropriate depth (as described in Table 1) to achieve an elicitation of needle sensation (the so-called “De Qi”), and the needles were retained for 20 minutes without any needle stimulation [18]. The acupuncture treatment was initiated on the tenth day after the first day of the LMP of each menstrual cycle and was repeated for three consecutive cycles. During each cycle, the participants were treated three times a week (Monday, Wednesday, and Friday) for two weeks to ensure that six sessions of acupuncture treatment were accomplished before the initiation of the next menstrual period. Thus, a total of 18 acupuncture sessions were scheduled during the three menstrual cycles. Acupuncture treatment was performed by a certified acupuncturist who was experienced in gynecological diseases.

### 2.5. Assessments

Five variables were measured: (1) maximal dysmenorrhea pain scores assessed by a NRS ranging from 0 to 10 with 0 described as no pain and 10 described as maximal pain; (2) number of days suffering from dysmenorrhea; (3) amount of rescue analgesic used for relief of dysmenorrhea; (4) quality of life assessed by the Short Form Health Survey (SF-36) questionnaire; (5) verbal multidimensional scoring system (VMSS) for assessment of dysmenorrhea evaluated from three domains including ability to work, systemic symptoms, and use of analgesics. VMSS included three grades: Grade 0, menstruation not painful and daily life activity unaffected; Grade 1, menstruation painful but seldom inhibiting normal activity, analgesics seldom required, and mild pain; Grade 2, daily activity affected, analgesics required and giving sufficient relief so that absence from school is unusual, and moderate pain; Grade 3, activity clearly inhibited, poor effect of analgesics, vegetative symptoms such as headache, fatigue, vomiting, and diarrhea, and severe pain.

Women self-rated the above measured variables as a baseline before beginning treatment and at the end of each menstrual cycle for three consecutive cycles, with an exception for quality of life which was self-rated at baseline and at the end of the study period. The primary outcome was the proportion (percentage) of responders in each of the two groups. Responders were defined as participants whose maximal dysmenorrhea pain scores (NRS) decreased at least 33% below their baseline [17]. The secondary outcomes included changes in the mean values of maximal dysmenorrhea pain scores, number of days suffering from dysmenorrhea, amount of rescue analgesic used for relief of dysmenorrhea, quality of life as assessed by the SF-36 questionnaire, and proportion of participants in each treatment group who experienced at least a one grade improvement in VMSS. Complete physical examinations and nondirective questioning regarding adverse events were accomplished at each follow-up visit to ensure patient safety.

### 2.6. Statistical Analysis

All participants who had received either at least one dose of the assigned COC or one session of acupuncture were included in the intention-to-treat analysis for efficacy and safety assessments. The last observation carried forward approach was used to analyze data of the participants who withdrew or who were lost to follow-up at any time after the assigned treatment had been initiated. *P* values of <0.05 were considered statistically significant. The analyses were done using STATA version 12.1.

For within-group comparison, one-way analysis of variance (ANOVA) with repeated measurement was used to determine the differences in the mean values of the measured variables (i.e., maximal dysmenorrhea pain scores, number of days per cycle suffering from dysmenorrhea, and amount of rescue analgesic used for relief of dysmenorrhea) between baseline and subsequent assessment points. Mean values of SF-36 scores at baseline and after completion of three treatment cycles in each group were compared using paired *t*-test.

For between-group comparison, the mean changes from baseline of variables mentioned above were compared using Student's *t*-test. The chi-square or Fisher's exact test was used to determine whether the two groups differed in response rate, withdrawal rate, or rate of loss to follow-up.

## 3. Results

**Figure 2:**
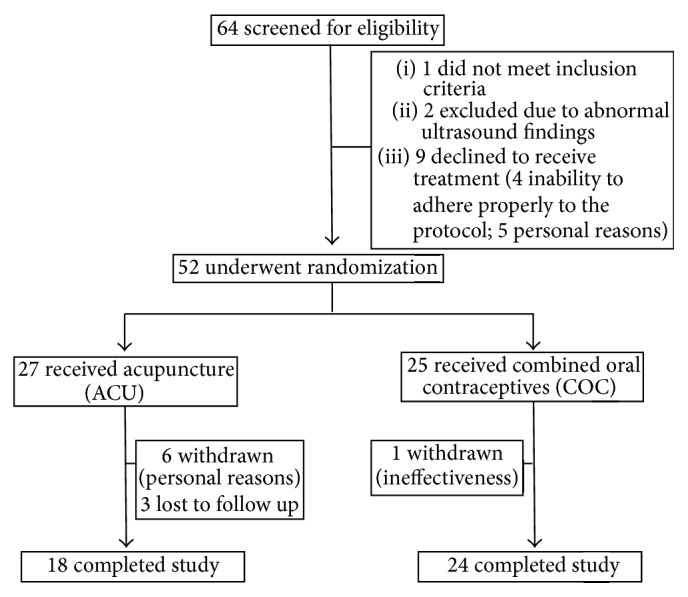
Study participation flow chart.

Sixty-four women were screened of whom 52 met the eligibility criteria. Those 52 were randomized to receive one of the assigned treatments (ACU, *n* = 27; COC, *n* = 25). There were no statistically significant differences in baseline characteristics between the two groups except for headache which was more prevalent in the ACU group (Table 2). At the end of the study, there were 18 completers in the ACU group and 24 in the COC group (Figure 2). The withdrawal rate and rate of loss to follow-up in the ACU group were significantly greater than those in the COC group (*P* = 0.012, Fisher's exact test).

Among the completers, treatment compliance in the COC group was determined based on the quantity of contraceptives remaining which had been prescribed at the previous visit, whereas treatment compliance in the ACU group was determined by the number of acupuncture sessions out of the total of 18 that participants had received. A “good compliance” was considered to be completion of more than 80% of an assigned treatment (COC or acupuncture) during the study period. Determination of treatment compliance found that all completers in each treatment group had a good compliance.

**Figure 3:**
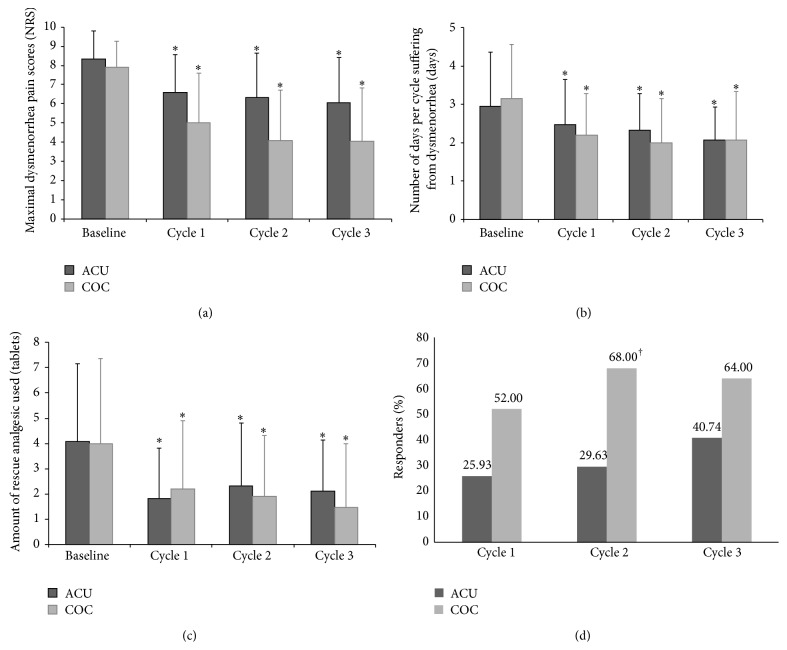
Measured variables in the acupuncture (ACU) group and the combined oral contraceptive (COC) pill group. (a) Mean maximal dysmenorrhea pain. (b) Mean number of days per cycle suffering from dysmenorrhea. (c) Mean amount of rescue analgesic used for relief of dysmenorrhea. (d) Percentage of responders. ^*∗*^ *P* < 0.05 compared with the respective baseline values (one-way ANOVA with repeated measurement). ^†^ *P* < 0.05 compared with ACU group at the respective time point (chi-square test).

In a within-group comparison, the mean values of outcome variables (maximal dysmenorrhea pain scores, number of days per cycle suffering from dysmenorrhea, and amount of rescue analgesic used for relief of dysmenorrhea) during each cycle in both groups significantly decreased from their respective baseline values (Figures 3(a)–3(c)). Evaluation of quality of life found that mean SF-36 scores after completion of three treatment cycles improved significantly from baseline in both the ACU group (from 85.30 ± 13.17 to 103.74 ± 17.99, *P* < 0.0001, paired *t*-test) and the COC group (from 91.72 ± 16.82 to 112.88 ± 14.28, *P* = 0.0001, paired *t*-test). Furthermore, both groups showed statistically significant improvement from baseline in physical functioning, role-physical and bodily pain, role-emotional, general health, vitality, and social functioning (data not shown).

A between-group comparison of primary outcomes found that the proportion of responders in the COC group increased, reaching a plateau at cycle 2, while the response rates in the ACU group gradually increased throughout the study. The response rate in the COC group at cycle 2 was statistically greater than that in the ACU group. There were no statistical differences between groups neither at the first cycle nor after completion of three treatment cycles (Figure 3(d)).

In a between-group comparison of secondary outcomes (Table 3), mean changes from baseline of maximal dysmenorrhea pain scores in the COC group at each cycle improved significantly compared with those in the ACU group. Nonetheless, mean changes from baseline of the remaining outcome variables (number of days per cycle suffering from dysmenorrhea, amount of rescue analgesic used for relief of dysmenorrhea, and quality of life assessed by SF-36 questionnaire) did not differ significantly between groups. Additionally, the percentage of participants who experienced an improvement in VMSS, defined as regression of VMSS of at least one grade from baseline, was comparable during the three treatment cycles in the ACU and COC groups (51.85% versus 56.00% at cycle 1, 44.44% versus 44.00% at cycle 2, and 44.44% versus 56.00% at cycle 3).

The majority of participants in the ACU and the COC groups experienced no adverse events (70.37% and 68.00%, *P* = 0.853, chi-square test). Among the 27 women in the ACU group, the most common adverse events were local irritation or minor bleeding at acupuncture points (15 events). Other adverse events included headache or myalgia (four events) and fever (one event). All adverse events were minor in intensity and completely self-limited. Among the 25 women in the COC group, the most common adverse event was abnormal vaginal bleeding (nine events). Other reported adverse events were headache or myalgia (five events), weight gain (three events), nausea or vomiting (two events), and breast engorgement (two events). No serious treatment side effects were found. There was no statistical difference in overall incidence of adverse events between the groups (*P* = 0.503, Fisher's exact test). However, it is noteworthy that the incidence of abnormal vaginal bleeding in the COC group was significantly higher than in the ACU group (36.0% versus 0%, *P* = 0.001, Fisher's exact test).

## 4. Discussion

In spite of the potential benefits to patients, the integration of complementary and alternative medicine (CAM) such as acupuncture into clinical practice based on western medicine is complicated in most areas of the world. In part, that is due to the general lack of knowledge and understanding of CAM in western medicine and due to the fact that the underlying rationale behind the two management systems appears, at least initially, to be contradictory.

This study found that both acupuncture and COC resulted in significant improvement in all outcome variables (maximal dysmenorrhea pain scores, number of days per cycle suffering from dysmenorrhea, amount of rescue analgesic used for relief of dysmenorrhea, and quality of life assessed by SF-36 questionnaire) compared with baseline. Most statistically significant benefits were demonstrated from the first cycle of treatment; however, over the three treatment cycles, COC was more efficacious than acupuncture with respect to reduction in maximal dysmenorrhea pain scores, whereas the efficacy of both interventions was comparable with respect to reduction in the number of days per cycle suffering from dysmenorrhea, reduction in rescue analgesic used for relief of dysmenorrhea, improvement in quality of life, and regression in VMSS score. Acupuncture had a lower rate of adherence to treatment than did COC. Adverse events with acupuncture were primarily related to minor irritation or minor bleeding at acupuncture point(s), while with COC the adverse events were hormone-related side effects such as abnormal vaginal bleeding, weight gain, nausea or vomiting, and breast engorgement.

To evaluate treatment of dysmenorrhea, many studies have used pain reduction or pain scores as a primary outcome. In addition, several related aspects such as additional analgesia required and absence from school or work have also been assessed [22]. In this study, a variety of assessments were included in order to more thoroughly evaluate treatment effects. Dysmenorrhea grading (VMSS) and quality of life were also included to provide a holistic evaluation of treatment efficacy.

It has been demonstrated that acupuncture can provide greater improvement in dysmenorrhea pain relief than a placebo control, NSAIDs, and Chinese herbs [12, 16, 17, 23–30]. Acupuncture point selection for treatment of various diseases, including primary dysmenorrhea, can be divided into two main approaches: individualized acupuncture and formula acupuncture. In individualized acupuncture, selection and combinations of acupuncture points are tailored to individual symptoms, signs, and syndrome differentiations as described in traditional Chinese medicine theory. In contrast, formula acupuncture includes a group of acupuncture points that are believed to provide therapeutic benefits for all patients without the necessity to establish a definite syndrome differentiation, so that the complicated diagnostic techniques, such as pulse and tongue diagnoses made by highly skilled traditional Chinese medical professionals, are not compulsory. Formula acupuncture was selected as the approach of choice for this study because it consists of a total of only six acupuncture points speculated to be thoroughly effective in rebalancing body homeostasis or eliminating pathologic factor such as cold, dampness, and damp-heat. The actions of the individual points used in this study are listed in Table 1. It could be expected that fewer needles should lead to lower physical and emotional burdens during treatment. Additionally, other studies have indicated that individualized acupuncture is not more effective than formula acupuncture in relieving symptoms in painful disorders [31, 32], giving further support for the use of formula acupuncture in this study. While needling, a needle sensation was elicited, ensuring that the needle punctures are exactly at the correct point. All needles were retained for 20 minutes without any additional stimulation because it has been demonstrated that although needle insertion leads to analgesia, needle stimulation is not crucial [33]. In addition, the superficial needling technique has also been demonstrated to be equally effective as the electrical needle stimulation technique in relieving dysmenorrhea [25]. During each cycle, six sessions of acupuncture treatment were scheduled before the initiation of the next menstrual period. That spacing of treatment was used because, according to the theory of traditional Chinese medicine, rebalancing of body homeostasis is an anticipated result of multiple sessions of acupuncture. Additionally, the cumulative and carryover effects of acupuncture analgesia (rather than the acute effects of acupuncture during a menstrual period) were the primary focus of this study. The three treatment cycles of acupuncture treatment in this study follow the appropriate duration recommended by other studies [16, 17].

This study demonstrated that acupuncture can significantly improve all measured outcomes over baselines. These findings are in accordance with other studies showing that acupuncture helps to reduce mean values of pain intensity, duration of menstrual pain, and need for additional analgesia and helps to improve quality of life [12, 15–17]. Nonetheless, the present study found a lower response rate in the ACU group (40.74% at cycle 3) compared with the value of 63.40% reported by Witt et al. [17]. This incongruity could be due to factors such as differences in age of the participants, ethnicity, acupuncture points, needle stimulation technique, frequency and duration of treatment, and so forth.

The synthetic hormones in COC cause a reduction in uterine prostaglandins [5] which leads to relief of dysmenorrhea. No significant differences among various COC preparations have been reported. Preparations of COC with ethinyl estradiol doses less than 35 mcg have been shown to be effective and are recommended as the preparation of choice [22]. As to the progestin component, a study reported more dysmenorrhea symptom relief using COC containing the third generation progestin than using COC containing the second generation progestin [10]. In this study, COC containing 20 *µ*g ethinyl estradiol and 150 *µ*g desogestrel, a third generation progestin, was used. By the end of the study, COC had resulted in significant improvement over baselines in all outcomes. Davis et al. previously demonstrated that COC containing 20 *µ*g ethinyl estradiol and 100 *µ*g levonorgestrel exerted a response rate of approximately 85% at the three-month evaluation in women with moderate-to-severe dysmenorrhea [13]; similarly, other studies have reported that various COC formulae relieved dysmenorrhea pain in up to 70–80% of women [7–10]. The aforementioned rates were somewhat higher than the 64% in the present study. Various factors (e.g., COC formula, age, ethnicity, and criteria for defining responders) might contribute to the different success rates of treatment with COC.

To the best of our knowledge, the relative efficacy of acupuncture and COC has not yet been compared. This study found that the response rate in the COC group had already attained a plateau by cycle 2, while that of the ACU group continued to increase gradually over time. The response rate in the COC group at cycle 2 was statistically greater than that of the ACU group; however, there were no statistical differences between groups after completion of three treatment cycles. This phenomenon suggests that the therapeutic effects of acupuncture are somewhat slower than those of COC. Nonetheless, it is worth noting that lack of statistical difference in response rates between the COC and ACU groups at cycle 3 (64.00% versus 40.74%) could have resulted from an inadequate power of test due to the small number of participants. Overall, acupuncture seems unlikely to be a suitable option in women who anticipate a faster therapeutic outcome. Nevertheless, this study did not determine whether or not the maximal effect from acupuncture had been achieved at the end of cycle 3. Further investigation over a longer study period should be undertaken.

Although this study found that acupuncture was less efficacious than COC in reducing maximal dysmenorrhea pain scores, its efficacy was comparable to COC in terms of improvement in the remaining outcome variables. Furthermore, acupuncture did not cause any hormone-related side effects as did COC. These findings support the potential use of acupuncture as an option in relieving dysmenorrhea, especially in some settings where COC is not considered to be a favorable choice such as in candidates who desire to get pregnant or those who experience intolerable adverse events as well as in cases where there are other contraindications or precautions related to COC, for example, undiagnosed abnormal genital bleeding, migraine with focal neurologic symptoms, thrombophlebitis or thromboembolic disorders, known or suspected estrogen-dependent neoplasia, and complicated valvular heart disease [34]. Additionally, the current findings are in accordance with a previous report showing that acupuncture significantly reduces the use of analgesia [16]. Since NSAIDs, frequently used as rescue analgesics, are well known to be associated with many side effects [35], reduction in the need for rescue NSAIDs (or other analgesics) leading to lower risks of adverse drug reactions is an additional benefit that could potentially be expected from acupuncture treatment.

The higher withdrawal rate and rate of loss to follow-up in the ACU group suggest that incomplete adherence to treatment might be in part due to the fact that acupuncture treatment is more time-consuming: women in the ACU group had to receive 18 sessions of acupuncture treatment at a clinic over a period of three months, whereas those in the COC group could take pills by themselves at home. Lower adherence to acupuncture treatment seems likely to be an important factor contributing to inferior therapeutic outcomes (less reduction in maximal dysmenorrhea pain scores) compared with COC, especially when an intention-to-treat analysis is used. Women who expect a favorable outcome from acupuncture treatment should, therefore, be strongly encouraged to maintain good compliance and cooperation.

Some limitations concerning this study should be mentioned. First, this study was designed as an open-blind study, so an awareness of the assigned treatment by participants and physician may have introduced bias. Second, only one acupuncture protocol was used. Different acupuncture protocols could conceivably result in different outcomes. Other variables that could possibly affect the therapeutic outcome of acupuncture include acupuncture point selection and combination, type of acupuncture (formula versus individualized), needle stimulation technique (manual manipulation, moxibustion, or electrical stimulation), and the number of acupuncture sessions per treatment cycle as well as frequency and duration of treatment. Further investigation with different protocols is needed. Third, the sample size used in this study was small, possibly leading to an inadequate power of test to identify any differences between the interventions. Fourth, the higher withdrawal rate and rate of loss to follow-up in the ACU group possibly could have resulted in an underestimation of acupuncture efficacy. Finally, as mentioned above, the three-month duration of the study was not long enough to determine whether or not maximal effect from acupuncture had been achieved by the end of the study. Double-blind trials using nonpenetrating placebo needles and with a larger sample size and a longer study period are warranted.

## 5. Conclusions

During three treatment cycles, COC was more efficacious than acupuncture with respect to reduction in maximal dysmenorrhea pain scores, whereas the efficacy of both interventions was comparable with respect to improvement in other outcome variables (number of days per cycle suffering from dysmenorrhea, amount of rescue analgesic used for relief of dysmenorrhea, quality of life, and VMSS score). At the end of the study, the response rate following both interventions did not statistically differ. Acupuncture commonly caused minor irritation or minor bleeding at the punctured point(s), while COC potentially caused hormone-related side effects. Acupuncture could be considered as potential alternative option in relieving moderate-to-severe dysmenorrhea, especially when COC is not a favorable choice.

## Figures and Tables

**Table 1:** Acupuncture points used for treatment of dysmenorrhea in this study [18].

Point	Location	Needling	Action
Qi Hai (Ren 6)	On the anterior midline, 1.5 cun inferior to the umbilicus or 3.5 cun superior to the upper border of the pubic symphysis	Vertically 0.8–1.5 cun	(i) Tonifies and regulates vital energy (ii) Resolves dampness

Zhong Ji (Ren 3)	On the anterior midline, 1 cun superior to the upper border of the pubic symphysis or 4 cun inferior to the umbilicus	Vertically 0.5–1 cun	(i) Resolves dampness and damp-heat (ii) Regulates menstruation (iii) Strengthens the kidneys

Di Ji (SP 8)	3 cun distal to the junction of the shaft and the medial condyle of the tibia, at the posterior border of the medial crest of the tibia	Vertically 1–1.5 cun	(i) Regulates the uterus (ii) Regulates vital energy and blood (iii) Stops menstrual pain by removing blood stasis

San Yin Jiao (SP 6)	3 cun proximal to the highest prominence of the medial malleolus, on the posterior border of the medial crest of the tibia	Vertically 1–1.5 cun	(i) Resolves dampness (ii) Promotes the function of the liver and the smooth flow of vital energy in the liver (iii) Tonifies the kidneys (iv) Nourishes blood and Yin (v) Regulates the uterus and menstruation (vi) Moves blood and eliminates blood stasis (vii) Cools blood (viii) Stops pain (ix) Calms the mind

The cun is a traditional Chinese unit of length equal to the width of a patient's thumb at the knuckle.

**Table 2:** Baseline characteristics of the study population in the acupuncture (ACU) and the combined oral contraceptive (COC) groups.

Variable	ACU	COC	*P* value
	(*n* = 27)	(*n* = 25)	
	Mean (SD)		
Age (years)	24.15 (6.0)	26.48 (4.4)	0.119
Age at menarche (years)	12.70 (1.5)	13.32 (1.2)	0.110
Duration of dysmenorrhea (years)	14.96 (3.3)	15.76 (2.7)	0.350
Number of dysmenorrhea days per cycle (days)	2.96 (1.4)	3.16 (1.4)	0.615
Maximal dysmenorrhea pain scores (NRS)	8.33 (1.5)	7.92 (1.4)	0.297
Rescue analgesic use (tablets per menstrual cycle)	4.07 (3.1)	4.00 (3.4)	0.934
Quality of life (SF-36) (score points)	85.30 (13.2)	91.72 (16.8)	0.130

	*n* (percent)		

Ability to work			0.693
Unaffected	0	1 (4.00)	
Rarely affected	5 (18.52)	5 (20.00)	
Moderately affected	12 (44.44)	12 (48.00)	
Significantly affected	10 (37.04)	7 (28.00)	
Associated symptoms			
None	0	2 (8.00)	0.134
Nausea	11 (40.74)	5 (20.00)	0.105
Vomiting	5 (18.52)	1 (4.00)	0.102
Headache	17 (62.96)	8 (32.00)	0.026
Diarrhea	8 (29.63)	10 (40.00)	0.432
Fatigue	23 (85.19)	16 (64.00)	0.078
Fever	3 (11.11)	1 (4.00)	0.336
Dizziness	10 (37.04)	7 (28.00)	0.488
Other	7 (25.93)	7 (28.00)	0.866
Verbal multidimensional scoring system (VMSS) grading			0.088
Grade 2	5 (18.52)	10 (40.00)	
Grade 3	22 (81.48)	15 (60.00)	

^*∗*^ *P* values are based on Student's *t*-test or chi-square test as appropriate. SD: standard deviation; *n*: number of individuals: NRS: numeric rating scale (0 to 10); SF-36: Short Form Health Survey.

**Table 3:** Mean (SD) change from baseline of outcome variables in intention-to-treat analysis between acupuncture (ACU) and combined oral contraceptive (COC) pill groups.

Outcome variable	Treatment group	Cycle 1-0	Cycle 2-0	Cycle 3-0
Maximal dysmenorrhea pain scores (NRS)	ACU	−1.74 (1.61)	−2.00 (1.88)	−2.30 (1.90)
	COC	−2.92 (2.44)^*∗*^	−3.84 (2.74)^*∗*^	−3.88 (2.52)^*∗*^

Number of days per cycle suffering from dysmenorrhea (days)	ACU	−0.48 (1.28)	−0.63 (1.39)	−0.89 (1.48)
	COC	−0.96 (1.30)	−1.16 (1.28)	−1.08 (1.32)

Amount of rescue analgesic used for relief of dysmenorrhea (tablets)	ACU	−2.26 (2.97)	−1.74 (3.25)	−1.96 (2.21)
	COC	−1.8 (3.92)	−1.28 (4.42)	−2.52 (3.04)

Quality of life assessed by SF36 (overall score)	ACU	ND	ND	12.38 (13.21)
	COC	ND	ND	14.20 (14.43)

^*∗*^Values statistically different from the ACU group (*P* < 0.05, Student's *t*-test). NRS: numeric rating scale; ND: not determined.
